# Surgical resection and survival outcomes in metastatic young adult colorectal cancer patients

**DOI:** 10.1002/cam4.3940

**Published:** 2021-06-16

**Authors:** Nina D. Arhin, Chan Shen, Christina E. Bailey, Lea K. Matsuoka, Alexander T. Hawkins, Andreana N. Holowatyj, Kristen K. Ciombor, Michael B. Hopkins, Timothy M. Geiger, Audrey E. Kam, Marc T. Roth, Cody M. Lebeck Lee, Michael Lapelusa, Arvind Dasari, Cathy Eng

**Affiliations:** ^1^ Division of Hematology and Oncology Department of Internal Medicine Vanderbilt University Medical Center Nashville TN USA; ^2^ Department of Surgery Department of Public Health Sciences Penn State College of Medicine Hershey PA USA; ^3^ Division of Surgical Oncology and Endocrine Surgery Department of Surgery Vanderbilt University Medical Center Nashville TN USA; ^4^ Division of Hepatobiliary Surgery and Liver Transplantation Department of Surgery Vanderbilt University Medical Center Nashville TN USA; ^5^ Section of Colon & Rectal Surgery Department of Surgery Vanderbilt University Medical Center Nashville TN USA; ^6^ Department of Medicine Vanderbilt University Medical Center/Vanderbilt‐Ingram Cancer Center Nashville TN USA; ^7^ Division of Hematology and Oncology Department of Internal Medicine Vanderbilt University Medical Center/Vanderbilt‐Ingram Cancer Center Nashville TN USA; ^8^ Division of General Surgery, Colon and Rectal Surgery Department of Surgery Vanderbilt University Medical Center Nashville TN USA; ^9^ Division of General Surgery, Colon and Rectal Surgery Department of Surgery Vanderbilt University Medical Center Nashville TN USA; ^10^ Division of Hematology, Oncology and Cell Therapy Department of Internal Medicine Rush University Medical Center Chicago IL USA; ^11^ Division of Hematology and Oncology Department of Internal Medicine Vanderbilt University Medical Center Nashville TN USA; ^12^ VA Medical Center Nashville TN USA; ^13^ Department of Internal Medicine Vanderbilt University Medical Center Nashville TN USA; ^14^ Department of Gastrointestinal Medical Oncology The University of Texas MD Anderson Cancer Center Houston TX USA; ^15^ Division of Hematology and Oncology Department of Internal Medicine Vanderbilt University Medical Center/Vanderbilt‐Ingram Cancer Center Nashville TN USA

**Keywords:** cancer, colorectal, overall survival, surgery, young adult

## Abstract

**Background:**

The incidence of colorectal cancer in adults younger than age 50 has increased with rates expected to continue to increase over the next decade. The objective of this study is to examine the survival benefit of surgical resection (primary and/or metastatic) versus palliative therapy in this patient population.

**Methods:**

We identified 6708 young adults aged 18–45 years diagnosed with metastatic colorectal cancer (mCRC) from 2004 to 2015 from the SEER database. Overall survival (OS) was analyzed using Kaplan–Meier estimation, log rank test, and multivariate Cox proportional hazards model.

**Results:**

Sixty‐three percent of patients in our study underwent primary tumor resection (PTR), with 40% undergoing PTR alone and 23% undergoing both resection of primary disease and metastasectomy. The median OS for patients who underwent both PTR and metastasectomy was 36 months, compared to 13 months for those who did not receive any surgical intervention. The multivariate analysis showed significant OS benefit of receiving both PTR and metastasectomy (HR 0.34, 95% CI: 0.31–0.37, *p* < 0.001) compared to palliative therapy. Undergoing PTR only and metastasectomy only were also associated with improved OS (HR 0.46, 95% CI: 0.43–0.49, *p* < 0.001 and HR 0.64, 95% CI: 0.55–0.76, *p* < 0.001, respectively).

**Conclusion:**

This is the largest observational study to evaluate survival outcomes in young‐onset mCRC patients and the role of surgical intervention of the primary and/or metastatic site. Our study provides evidence of statistically significant increase in OS for young mCRC patients who undergo surgical intervention of the primary and/or metastatic site.

## Graphical abstract

The incidence of colorectal cancer in adults younger than age 50 has increased with rates expected to continue to increase over the next decade. The objective of this study is to examine the survival benefit of surgical resection (primary and/or metastatic) vs palliative therapy in this patient population. Our study provides evidence of statistically significant increase in OS for young mCRC patients who undergo surgical intervention of the primary and/or metastatic site.

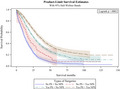

## 1 INTRODUCTION

Colorectal cancer (CRC) is the third leading cause of cancer death in the United States, and the second leading cause of cancer death worldwide.^1^, ^2^ Though the incidence of colorectal cancer in adults age 50 or older is declining partly due to increasing awareness and availability of screening tests such as fecal screening tools, endoscopic screenings and CT colonography, the incidence of colorectal cancer in adults younger than age 50 has continued to increase.^3^, ^4^, ^5^ Young adults with colorectal cancer commonly present with advanced stage disease, higher pathological grading, poorly differentiated histology, higher cases of signet ring cell histology, and left‐sided primary tumors.^6^, ^7^

Some specific risk factors pertaining to early‐onset colorectal cancer include inflammatory bowel disease which leads to a two‐ to threefold increased risk, hereditary cancer predisposing syndromes, familial CRC syndromes, and history of abdominal irradiation.^7^, ^8^, ^9^, ^10^ Approximately 30% of young adults with colorectal cancer have mutations that cause hereditary cancer syndromes, 20% have familial colorectal cancer with the remaining 50% having neither hereditary nor familial colorectal cancer.^7^ The majority of early‐onset colorectal cancer is considered sporadic, with the exact etiology being considered to be multifactorial in the setting of environmental and behavioral changes as well as alterations in the microbiome.^7^ Lieu et al. (2019) discovered that tumors from younger and older patients with colorectal cancer demonstrated similar overall rates of genomic alteration.^11^ However, Holowatyj et al. (2020) among others have shown that early‐onset colorectal cancer harbors distinct biology including more genetic variants and distinct molecular phenotype indicating the need for further studies to determine the exact etiology of early‐onset colorectal cancer.^12^

In 2020, it is expected that 12% of colorectal cancer cases (about 18,000) will be diagnosed in adults under the age of 50.^13^ Based on the analysis by Bailey et al. (2015), it is expected that the incidence rate for colon cancer will increase by 90.0% and by 124.2% for rectosigmoid and rectal cancers for adults aged 20–34 by the year 2030.^5^ This suggests that 10% of all colon cancers and 22% of all rectal cancers will be diagnosed in patients under 50 years of age by 2030 compared to 4.8% and 9.5% in 2010, respectively.^7^

About 20% of all patients with colorectal cancer present with metastatic disease at the time of diagnosis.^14^ Yang et al. (2016) evaluated the long‐term effects of palliative interventions in metastatic colorectal cancer and discovered that, resection of oligometastatic disease led to a significant improvement in the 2‐, 3‐, and 5‐ year overall survivals in these patients (78%, 52.2%, and 26.2%, respectively) compared to patients who received chemotherapy alone (37%, 22%, and 11%, respectively).^15^ Despite the association between oligometastatic disease resection and long‐term survival benefit, most patients do not qualify for this approach, presumably due to the initial presentation with multiple sites of distant disease. But for those that are able to undergo surgical resection, it is likely attributable to community biases and availability of institutions capable of performing complex metastatic surgical resection of the primary tumor and distant sites of disease.^16^, ^17^

The objective of this study is to compare the overall survival of young adults aged 18–45 with metastatic colorectal cancer who underwent surgical resection of the primary tumor, oligometastatic resected disease or both versus those patients who received palliative chemotherapy. This is the largest study utilizing the SEER database to evaluate survival outcomes in young‐onset metastatic colorectal cancer patients and the role of palliative interventions in this patient population. Treatment patterns and outcomes of metastatic colorectal cancer in young adults are not well studied and this study aims to fill this knowledge gap.

## 2 METHODS

### 2.1 Data source and study cohort

We identified primary colorectal cancer cases diagnosed (not at autopsy) between years 2004 and 2015 from the Surveillance, Epidemiology, and End Results (SEER) registry data by the National Cancer Institute (NCI) (https://seer.cancer.gov/).^18^ The SEER cancer registry database is a well‐accepted data source for cancer epidemiology studies involving 34.6% of the US population. The SEER 18 custom data with additional treatment fields that we used provide detailed information on tumor characteristics, demographic information, treatment received, and survival.

We restricted the sample to young adults between 18 and 45 years of age diagnosed with AJCC 6th edition stage IV disease, and excluded cases with missing information on treatment received and survival. We considered patients’ characteristics including age at diagnosis, sex, race (White, Black, and other), indicator of Hispanic origin (Spanish‐Hispanic‐Latino, non‐Spanish‐Hispanic‐Latino), marital status (single, married, and other), primary tumor site (right‐sided, left‐sided, and undetermined), histology (adenocarcinoma not otherwise specified (NOS), mucinous adenocarcinoma, signet ring cell carcinoma, and other), year of diagnosis (2004–2007, 2008–2011, and 2012–2015), surgical treatment (both primary tumor resection (PTR) and metastasectomy, primary tumor resection without metastasectomy, metastasectomy without primary tumor resection, either primary tumor resection or metastasectomy), chemotherapy (yes, no/unknown), radiation (yes, no/unknown), and overall survival.

### 2.2 Statistical Analysis

We provide sample descriptive statistics for the study cohort. Kaplan–Meier estimation and log rank test were used to examine survival differences by treatment groups. We also employed multivariate Cox proportional hazard model to examine the association between treatment received and overall survival of the patients controlling for other patient characteristics. Hazard ratio (HR), 95% confidence interval (CI), and *p*‐value are provided. All statistical testes were two‐sided tests, results are considered statistically significant when *p* < 0.05. All statistical analyses were conducted in SAS 9.4.

## 3 RESULTS

Table 1 includes sample descriptives of this study. A total of 6,708 young adults age from 18 to 45 years diagnosed with metastatic colorectal cancer from 2004 to 2015 were identified from SEER and included in the study. The median age at diagnosis was 40.0 years, while the interquartile range was 36–43 years. Three thousand four hundred and forty‐five patients (51.4%) were male, 3,263 patients (48.6%) were female and 1,798 (26.8%) were non‐White. One thousand two hundred and eight patients (18%) were of Hispanic origin. The majority of patients (64.8%) had left‐sided tumors. Two thousand six hundred and fifty‐three patients (39.5%) underwent primary tumor resection alone, 1,547 (23.1%) underwent both primary tumor resection and metastasectomy, 231 (3.4%) received metastasectomy without primary tumor resection, and 2,277 patients (32.9%) did not undergo any surgical resection. Five thousand five hundred and fifty‐two patients (82.8%) received systemic chemotherapy, and 1,172 (17.2%) received radiation therapy. In regard to histology, 4786 patients (71.3%) had adenocarcinoma NOS, 669 (10%) had mucinous adenocarcinoma, and 287 (4.3%) had signet ring cell adenocarcinoma.

**TABLE 1:** Characteristics of the sample from SEER registry data

	N(%)
Total	6708 (100%)
Age at diagnosis	
N	6708
Mean (SD)	38.8 (5.77)
Median	40
Interquartile range	36.0, 43.0
Range	(18.0–45.0)
Sex	
Male	3445 (51.4%)
Female	3263 (48.6%)
Race	
White	4910 (73.2%)
Black	1040 (15.5%)
Other	758 (11.3%)
Hispanic Origin	
Non‐Spanish‐Hispanic‐Latino	5500 (82%)
Spanish‐Hispanic‐Latino	1208 (18%)
Marital Status	
Single	2264 (33.8%)
Married	3507 (52.3%)
Other	937 (14%)
Primary site	
Right‐sided	2026 (30.2%)
Left‐sided	4344 (64.8%)
Undetermined	338 (5%)
Histology	
Adenocarcinoma, NOS	4786 (71.3%)
Mucinous adenocarcinoma	669 (10%)
Signet ring cell carcinoma	287 (4.3%)
Other	966 (14.4%)
Year of diagnosis	
2004–2007	1970 (29.4%)
2008–2011	2254 (33.6%)
2012–2015	2484 (37%)
Primary Surgery	
Yes	4200 (62.6%)
No	2508 (37.4%)
Non‐Primary Surgery	
Yes	1778 (26.5%)
No	4930 (73.5%)
Types of Surgeries	
Yes PS + Yes NPS	1547 (23.1%)
Yes PS + No NPS	2653 (39.5%)
No PS + Yes NPS	231 (3.4%)
No PS + No NPS	2277 (33.9%)
Radiation	
Yes	1156 (17.2%)
No/Unknown	5552 (82.8%)
Chemotherapy	
Yes	5536 (82.5%)
No/Unknown	1172 (17.5%)

Abbreviations: NPS, Non‐primary surgery; PS, Primary Surgery.

**FIGURE 1:**
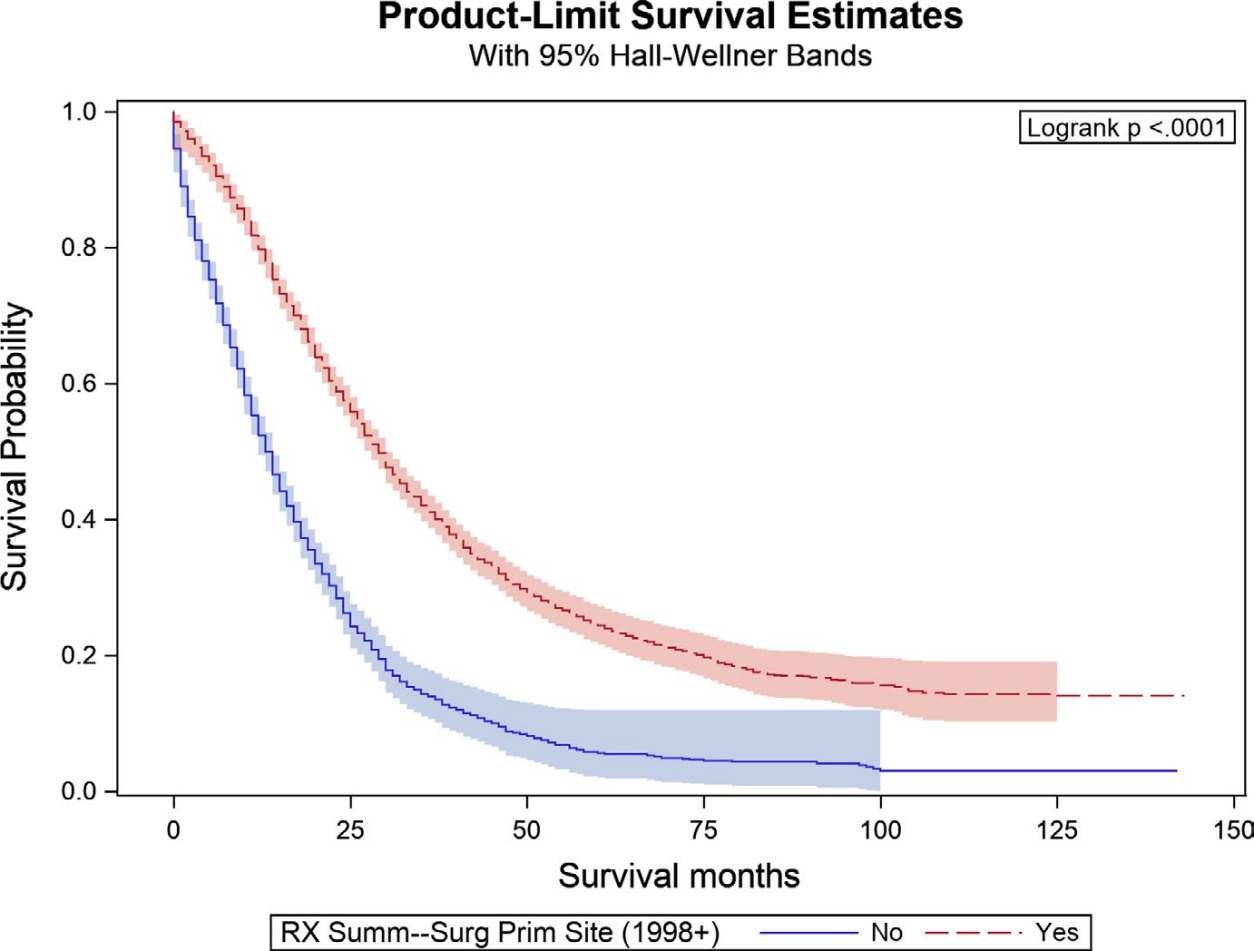
Kaplan‐Meier curves by primary surgery in the study cohort

**FIGURE 2:**
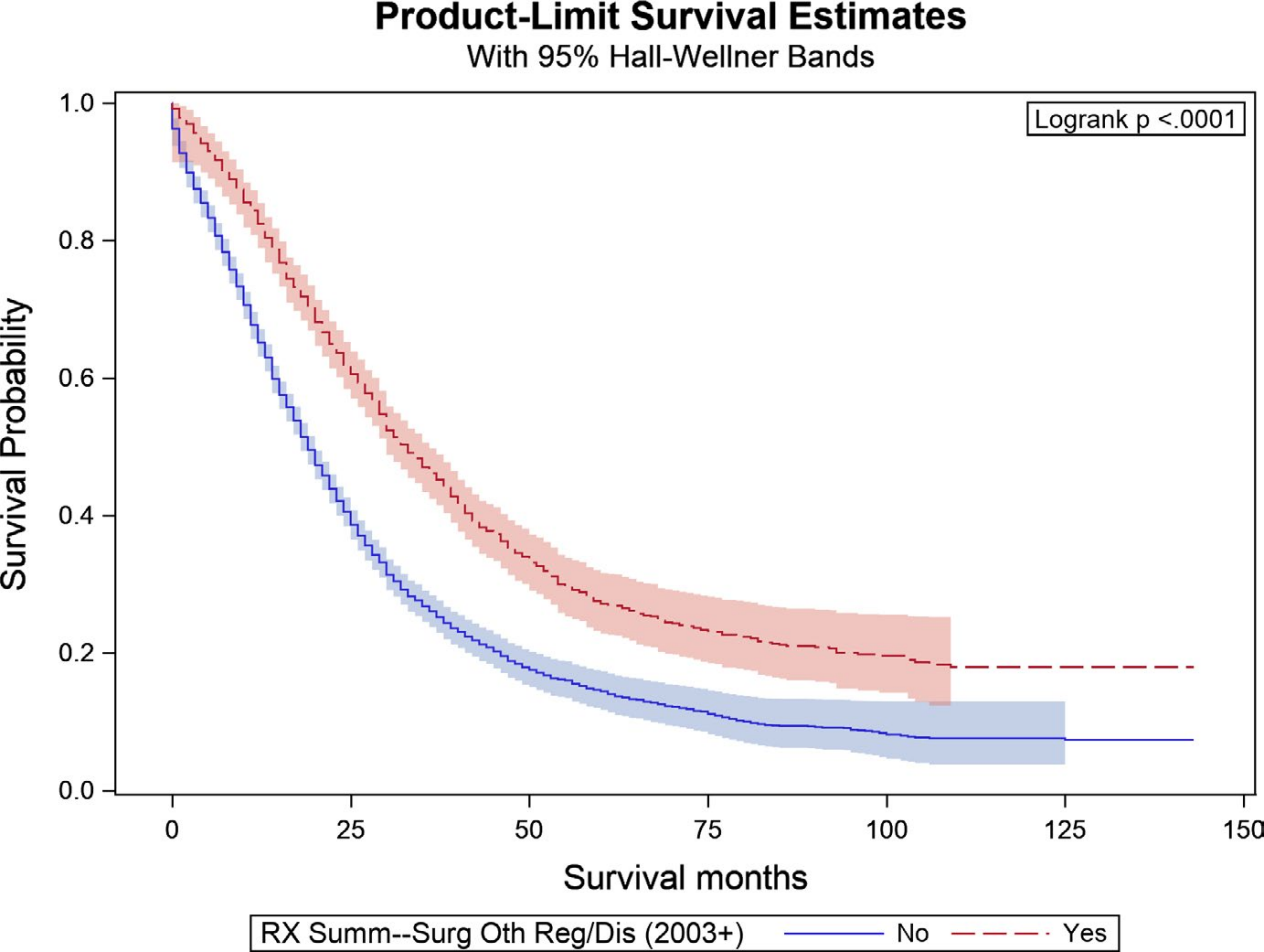
Kaplan‐Meier curves by non‐primary surgery in the study cohort

**FIGURE 3:**
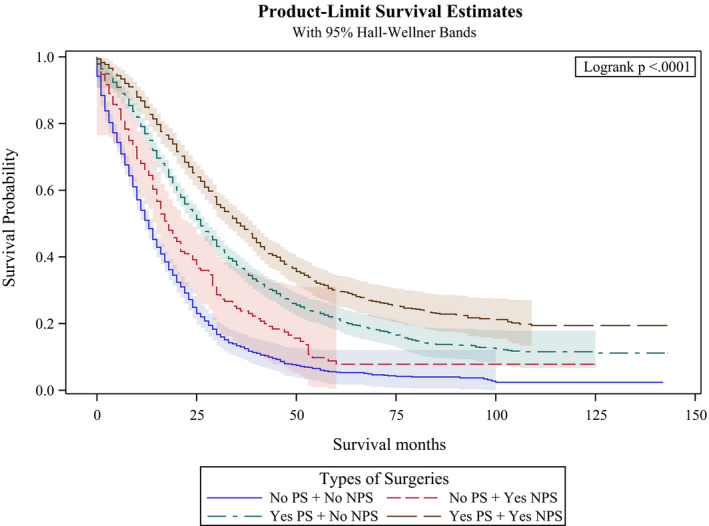
Kaplan‐Meier curves by type of surgery in the study cohort. PS: Primary Surgery; NPS: Non‐primary surgery

Figures 1, 2, and 3 provide the results from the OS analyses. The median OS of the whole cohort was 22 months while the interquartile range was 10–45 months. The KM curves in Figure 1 showed significant differences in the OS between patients who received primary tumor resection and those who did not (log rank test *p* < 0.001). The median OS for patients who underwent resection of their primary tumor was 29 months compared to 13 months for those who did not undergo primary tumor resection (log rank test *p* < 0.001). When we compare patients who received non‐primary site resection versus those who did not in Figure 2, we again observed a very significant difference in OS (log rank test *p* < 0.0001). The median OS for patients who underwent non‐primary site resection was 33 months compared to 19 months in those who did not (log rank test *p* < 0.001).

When we compared the four groups of patients (primary tumor resection alone, both primary tumor resection and metastasectomy, metastasectomy alone, or no surgical intervention), the KM curves in Figure 3 again showed significant survival differences (log rank test *p* < 0.0001). The median OS was longest for patients who underwent both primary tumor resection and metastasectomy at 36 months, followed by those who underwent primary tumor resection only at 26 months. Those who underwent metastasectomy alone had a median OS of 18 months, while patients who did not receive any surgical intervention had the shortest OS at 13 months.

Table 2 includes the results from the multivariate Cox proportional hazard model. The multivariate Cox proportional hazard model confirmed that compared to patients who did not undergo any surgical resection, patients who underwent both primary tumor resection and metastasectomy had the lowest hazard ratio of 0.34 (95% CI: 0.31–0.37, *p* < 0.001), followed by those who underwent primary tumor resection only (HR 0.46, 95% CI: 0.43–0.49, *p* < 0.001), and finally, those who underwent metastasectomy alone (HR 0.64, 95% CI: 0.55–0.76, *p* < 0.001). We also found that receiving chemotherapy and radiation therapy were significantly associated with better survival (HR 0.65, 95% CI: 0.60–0.70, *p* < 0.001 and HR 0.88, 95% CI: 0.81–0.95, *p* = 0.001, respectively). Improved overall survival was observed in patients diagnosed in more recent years (2008–2011 HR 0.89, 95% (CI): 0.83–0.95, *p* < 0.001; 2012–2015 HR 0.82, 95% (CI): 0.76–0.89, *p* < 0.001 compared to 2004–2007). Worse overall survival was observed in patients with right‐sided tumors compared to left‐sided tumors (HR 1.22, 95% (CI): 1.13–1.31, *p* < 0.001), males compared to females (HR 1.09, 95% (CI): 1.03–1.15, *p* = 0.004), Black compared to White (HR 1.21, 95% (CI): 1.12–1.31), *p* < 0.001), and single compared to married (HR 1.20, 95% (CI): 1.12–1.28), *p* < 0.001).

**TABLE 2:** Results from Cox proportional hazards model

	Hazard Ratio	95% CI	*p*‐value
Age at diagnosis	1	[0.99,1.00]	0.081
Sex			
Female	Reference		
Male	1.09	[1.03,1.15]	0.004
Race			
White	Reference		
Black	1.21	[1.12,1.31]	<0.001
Other	0.97	[0.88,1.06]	0.479
Hispanic Origin			
Non‐Spanish‐Hispanic	Reference		
Spanish‐Hispanic‐Latino	0.92	[0.85,1.00]	0.038
Marital Status			
Married	Reference		
Other	1.07	[0.98,1.17]	0.114
Single	1.2	[1.12,1.28]	<0.001
Primary site			
Left‐sided	Reference		
Right‐sided	1.22	[1.13,1.31]	<0.001
Undetermined	1.4	[1.24,1.60]	<0.001
Histology			
Adenocarcinoma, NOS	Reference		
Mucinous adenocarcinoma	0.81	[0.73,0.90]	<0.001
Other	1.03	[0.95,1.12]	0.481
Signet ring cell carcinoma	1.62	[1.41,1.85]	<0.001
Year of diagnosis			
2004–2007	Reference		
2008–2011	0.89	[0.83,0.95]	<0.001
2012–2015	0.82	[0.76,0.89]	<0.001
Types of Surgeries			
No PS + No NPS	Reference		
No PS + Yes NPS	0.64	[0.55,0.76]	<0.001
Yes PS + No NPS	0.46	[0.43,0.49]	<0.001
Yes PS + Yes NPS	0.34	[0.31,0.37]	<0.001
Radiation			
No/Unknown	Reference		
Yes	0.88	[0.81,0.95]	0.002
Chemotherapy			
No/Unknown	Reference		
Yes	0.65	[0.60,0.70]	<0.001

Abbreviations: NPS, Non‐primary surgery; PS, Primary Surgery.

**FIGURE 4:**
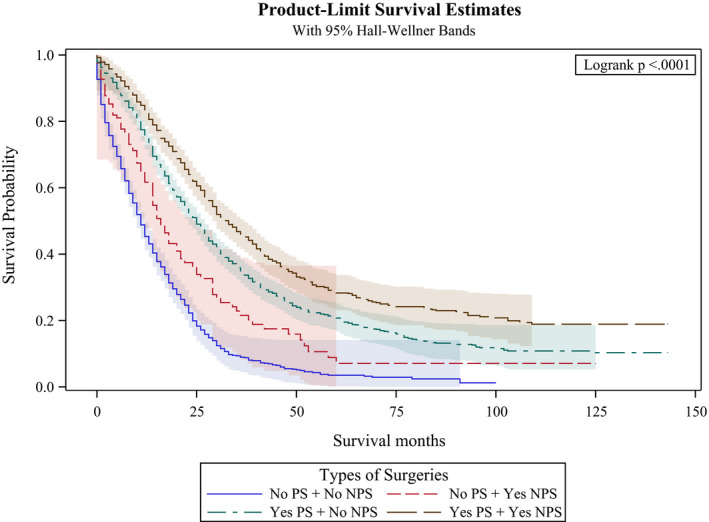
Kaplan‐Meier curves by type of surgery in the subcohort of patients with colon cancer. PS: Primary Surgery; NPS: Non‐primary surgery

**FIGURE 5:**
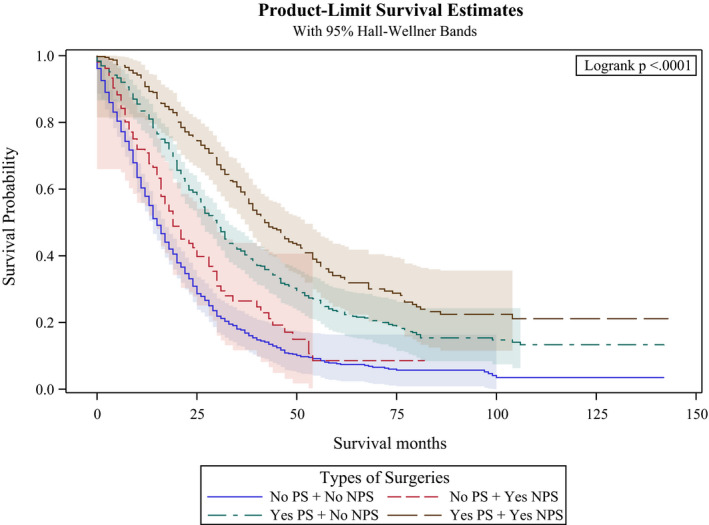
Kaplan‐Meier curves by type of surgery in the subcohort of patients with rectum cancer. PS: Primary Surgery; NPS: Non‐primary surgery

It is possible that patients who received primary tumor resection had better prognosis and were more likely to receive aggressive chemotherapy and radiation therapy. To deal with such potential bias, we conducted a sensitivity analysis using inverse probability of treatment weighting based on the propensity score. The results from this sensitivity analysis revealed similar results in terms of lower hazard ratios (HR 0.49, 95% (CI): 0.46–0.53, *p* < 0.001) for patients who underwent primary tumor resection. The detailed results are included in Table A1. Figures 4 and 5 provide sub‐analysis of colon and rectal carcinoma separately which revealed the same results in regards to overall survival months. Tables 3 and 4 include results from the multivariate Cox proportional hazard model which also revealed similar hazard ratios for patients with colon cancer that underwent primary tumor resection and metastasectomy (HR 0.43, 95% (CI): 0.40–0.47, *p* < 0.001) and HR 0.74, 95% (CI): 0.68–0.80, *p* < 0.001) compared to patients with rectal cancer that underwent primary tumor resection and metastasectomy (HR 0.49, 95% (CI): 0.44–0.54, *p* < 0.001) and HR 0.7, 95% (CI): 0.61–0.81, *p* < 0.001).

**TABLE 3:** Results from Cox proportional hazards model in the subcohort with colon cancer

	Hazard Ratio	95% CI	*p*‐value
Age at diagnosis	1	[0.99,1.00]	0.406
Sex			
Female	Reference		
Male	1.12	[1.04,1.20]	0.002
Race			
White	Reference		
Black	1.23	[1.12,1.35]	<0.001
Other	0.98	[0.88,1.11]	0.789
Hispanic Origin			
Non‐Spanish‐Hispanic	Reference		
Spanish‐Hispanic‐Latino	0.95	[0.86,1.05]	0.31
Marital Status			
Married	Reference		
Other	1.11	[0.99,1.23]	0.07
Single	1.21	[1.12,1.31]	<0.001
Histology			
Adenocarcinoma, NOS	Reference		
Mucinous adenocarcinoma	0.79	[0.71,0.89]	<0.001
Other	1.02	[0.92,1.14]	0.679
Signet ring cell carcinoma	1.6	[1.37,1.87]	<0.001
Year of diagnosis			
2004–2007	Reference		
2008–2011	0.86	[0.79,0.93]	<0.001
2012–2015	0.8	[0.72,0.88]	<0.001
Types of Surgeries			
No PS + No NPS	Reference		
No PS + Yes NPS	0.6	[0.48,0.75]	<0.001
Yes PS + No NPS	0.42	[0.38,0.46]	<0.001
Yes PS + Yes NPS	0.32	[0.29,0.35]	<0.001
Radiation			
No/Unknown	Reference		
Yes	1.15	[0.99,1.34]	0.071
Chemotherapy			
No/Unknown	Reference		
Yes	0.65	[0.60,0.71]	<0.001

Abbreviations: NPS, Non‐primary surgery; PS, Primary Surgery.

**TABLE 4:** Results from Cox proportional hazards model in the subcohort with rectum cancer

	Hazard Ratio	95% CI	*p*‐value
Age at diagnosis	0.99	[0.99,1.00]	0.21
Sex			
Female	Reference		
Male	1.03	[0.93,1.14]	0.572
Race			
White	Reference		
Black	1.22	[1.05,1.41]	0.008
Other	0.89	[0.76,1.05]	0.183
Hispanic Origin			
Non‐Spanish‐Hispanic	Reference		
Spanish‐Hispanic‐Latino	0.87	[0.75,0.99]	0.04
Marital Status			
Married	Reference		
Other	1.02	[0.88,1.18]	0.795
Single	1.19	[1.06,1.33]	0.003
Histology			
Adenocarcinoma, NOS	Reference		
Mucinous adenocarcinoma	1.41	[1.12,1.77]	0.004
Other	1.17	[1.02,1.35]	0.026
Signet ring cell carcinoma	1.9	[1.46,2.47]	<0.001
Year of diagnosis			
2004–2007	Reference		
2008–2011	0.95	[0.85,1.07]	0.43
2012–2015	0.87	[0.76,1.00]	0.05
Types of Surgeries			
No PS + No NPS	Reference		
No PS + Yes NPS	0.74	[0.58,0.95]	0.016
Yes PS + No NPS	0.49	[0.44,0.55]	<0.001
Yes PS + Yes NPS	0.34	[0.29,0.40]	<0.001
Radiation			
No/Unknown	Reference		
Yes	0.83	[0.75,0.93]	0.001
Chemotherapy			
No/Unknown	Reference		
Yes	0.59	[0.51,0.68]	<0.001

Abbreviations: NPS, Non‐primary surgery; PS, Primary Surgery.

## 4 DISCUSSION

Our study represents one of the largest outcomes analyses of young adults with metastatic colorectal cancer to date, with over 6,700 patients included. Sixty‐three percent of the patients in our study underwent primary tumor resection, with 40% undergoing primary tumor resection alone and 23% undergoing both resection of their primary disease and metastasectomy. The median overall survival (mOS) was significantly greater in both surgical groups compared to patients who did not undergo any surgical resection. Only 3% of the patients underwent metastasectomy without primary tumor resection and appeared to have higher mOS compared to patients who were not surgically resected. However, given the smaller percentage of the metastasectomy only group, it is underpowered to draw any definitive conclusions, but would be of interest for further evaluation. This study also supports previous reported data showing a higher incidence of left‐sided tumors in young adults presenting with colorectal cancer.

Previous studies evaluating surgical outcomes in all patients regardless of age with metastatic colorectal cancer have shown mixed results. Temple et al. (2004) and Cook et al (2005) performed large SEER data review in adults with metastatic colorectal cancer diagnosed from 1991 to 1999 and 1988 to 2000, respectively and discovered that primary tumor resection was performed in the majority of asymptomatic patients. A significant difference in the median overall survival was observed at 10–11 months in those who underwent cancer‐directed surgery compared with 2–3 months in those who did not.^19^, ^20^ Furthermore, several studies performed in Europe have also shown a clear benefit in the survival of patients who undergo resection of both primary tumor and metastatic disease compared to those who did not.^21^, ^22^, ^23^ Siebenhuner et al. (2020) performed a recent SEER review evaluating adult patients with diagnosis of metastatic colorectal cancer from 2010–2015 who underwent resection of primary tumor and metastatic disease. It revealed a significant difference in overall survival with a mOS of 31.2 months in those who underwent resection of their disease (primary tumor and metastases) compared to 20.4 months in those who did not.^24^ In contrast, Poultsides et al. (2009) revealed no improvement in overall survival in patients who underwent resection of primary tumor compared to those who did not.^25^ Preliminary results from the ongoing randomized phase III iPACS JCOG1007 trial also revealed no survival advantage for patients who undergo resection of their primary tumor compared to those who receive chemotherapy alone.^26^ The prospective nature of this randomized control trial will better address this question given the severe selection bias of retrospective studies. Given these conflicting results, additional prospective randomized studies are necessary to fully answer the question of survival benefit in this setting.

While prospective randomized trials are necessary to fully answer the question of a survival benefit in the metastatic setting,^16^ prior studies such as this study by Gulack et al. (2016) suggest that surgical intervention of the primary tumor has a palliative role in treatment outcomes.^27^ It is worthy to note that although, surgical intervention has been shown to provide a survival advantage in metastatic colorectal cancer, radiation therapy to oligometastatic sites has become an option available especially to patients with metastatic disease who are otherwise not good surgical candidates. However, the survival advantage of radiation therapy is an area for further exploration.^28^, ^29^

One limitation of our study is that the reason for surgical resection is not available in the SEER database. We therefore, do not have data as to the intent of surgery (palliation vs cure) or other factors influencing the interplay of surgery, chemotherapy, and radiation such as burden of disease, comorbidities, treatment location, symptom control (bowel obstruction, bleeding, peritonitis, and sepsis), or previous surgeries. Furthermore, we suspect selection bias may have contributed to the decrease in the mOS of patients who did not undergo surgical intervention as some may not have been surgical candidates due to several reasons such extensive metastatic disease, poor performance status etc. We also suspect that those who underwent primary tumor resection and metastasectomy likely had oligometastatic or resectable disease compared to those who only received palliative chemotherapy as the latter may have had higher tumor burden or not convertible to resectability leading to inherently better prognosis in the former. Moreover, we are unable to differentiate between patients with synchronous versus asynchronous metastases as the prognosis for patients with synchronous metastases is worse than those with asynchronous metastases.^30^ The OS of 13 months in patients who do not undergo PTR may reflect data from patients diagnosed with metastatic colorectal cancer in the early 2000 s, prior to the wide availability of the different chemotherapy and biologic therapies. Current data from clinical trials report an OS 30 to 37 months in patients with metastatic colorectal cancer treated with chemotherapy. Of note, we are unable to determine if patients who did not undergo PTR received or did not receive chemotherapy in SEER. It could be that patients that were unfit for surgery were possibly also unfit to receive chemotherapy leading to the lower OS in those who did not undergo PTR.^31^, ^32^ Though there have been other SEER studies showing survival benefit of primary tumor resection, our study is different as it focuses on young adults only.^33^, ^34^

In the management of metastatic colorectal cancer, molecular markers play an important role as they guide treatment choices and help to predict prognosis. The KRAS, the most commonly mutated RAS oncogene occurs in about 45% of colorectal cancers with other rare RAS mutations representing an additional 15% of patients.^35^ It is well documented that patients with a RAS mutation have reduced OS likely due to limited treatment options and relatively worse survival following liver resection.^35^, ^36^ The BRAF mutation occurs in about less than 10% of metastatic colorectal cancers and confers a poor prognosis.^37^, ^38^ Mismatch repair protein deficiency/high levels of microsatellite instability occurs in about 5% of metastatic colorectal cancer and was also regarded as a poor prognostic marker prior to the FDA approval of immunotherapy in this setting which resulted in a marked prolongation of OS of these selected patients.^38^, ^39^ Another limitation of this study is that these molecular markers are not routinely available in SEER and therefore, we are unable to differentiate between patients whose prognosis were affected by the presence of these mutations. Despite these limitations, young adults with metastatic colorectal cancer represent a unique patient population with growing incidence and our data suggest that pursuing aggressive surgical intervention for these patients may lead to improved overall outcomes.

## 5 CONCLUSION

Based on our study, surgical resection of the primary tumor and metastatic disease was associated with the best overall survival outcome for young metastatic colorectal cancer patients. As reasons for primary tumor resection vary across different patient populations, we believe a multidisciplinary approach should be considered in all young adults who maybe appropriate candidates for surgical resection. Surgery in the face of unresectable disease is sometimes important not only for palliation, but for potentially increasing survival. We believe this approach is essential, especially given the expected rise in the incidence of colorectal cancer among this patient population over the next decade. It is important for patients to understand the different treatment options available to them, and for clinicians to know when to refer for consideration for multidisciplinary treatment.

## CONFLICT OF INTEREST STATEMENT

The authors declare they have no conflict of interests or disclosures.

## ETHICS STATEMENT

Approval was waived by the local ethics committee, as SEER data is publicly available and de‐identified.

## Data Availability

The data sets generated for this study are available in the SEER database (https://seer.cancer.gov/about/overview.html).

## 1

**TABLE A1:** Sensitivity analysis results from Cox proportional hazards model using inverse probability of treatment weighting based on the propensity score for primary surgery

	Hazard Ratio	95% CI	p‐value
Age at diagnosis	1	[0.99,1.00]	0.142
Sex			
Female	Reference		
Male	1.09	[1.01,1.17]	0.023
Race			
White	Reference		
Black	1.18	[1.07,1.30]	0.001
Other	0.93	[0.83,1.05]	0.273
Hispanic Origin			
Non‐Spanish‐Hispanic	Reference		
Spanish‐Hispanic‐Latino	0.92	[0.83,1.03]	0.153
Marital Status			
Married	Reference		
Other	1.09	[0.98,1.20]	0.104
Single	1.2	[1.10,1.30]	<0.001
Primary site			
Left‐sided	Reference		
Right‐sided	1.18	[1.09,1.28]	<0.001
Undetermined	1.31	[1.11,1.55]	0.002
Histology			
Adenocarcinoma, NOS	Reference		
Mucinous adenocarcinoma	0.74	[0.62,0.87]	<0.001
Other	1.05	[0.93,1.17]	0.435
Signet ring cell carcinoma	1.52	[1.30,1.78]	<0.001
Year of diagnosis			
2004–2007	Reference		
2008–2011	0.91	[0.84,0.99]	0.036
2012–2015	0.84	[0.77,0.92]	<0.001
Primary Surgery			
No	Reference		
Yes	0.49	[0.46,0.53]	<0.001
Non‐Primary Surgery			
No	Reference		
Yes	0.69	[0.62,0.76]	<0.001
Radiation			
No/Unknown	Reference		
Yes	0.89	[0.81,0.97]	0.008
Chemotherapy			
No/Unknown	Reference		
Yes	0.68	[0.61,0.77]	<0.001

Abbreviations: NPS, Non‐primary surgeryPS, Primary Surgery.

## References

[1] American Cancer Society . Colorectal Cancer facts & figures 2020–2022. Atlanta: American Cancer Society; 2020. https://www.cancer.org/research/cancer‐facts‐statistics/colorectal‐cancer‐facts‐figures.html.

[2] Bray F , Ferlay J , Soerjomataram I , Siegel RL , Torre LA , Jemal A . Global cancer statistics 2018: GLOBOCAN estimates of incidence and mortality worldwide for 36 cancers in 185 countries. CA Cancer J Clin. 2018;68:394‐424. 30207593 10.3322/caac.21492

[3] Murphy CC , Sandler RS , Sanoff HK , Yang YC , Lund JL , Baron JA . Decrease in incidence of colorectal cancer among individuals 50 years or older after recommendations for population‐based screening. Clin Gastroenterol Hepatol. 2017;15(903–909):e906. 10.1016/j.cgh.2016.08.037 PMC5337450 27609707

[4] Davis DM , Marcet JE , Frattini JC , Prather AD , Mateka JJ , Nfonsam VN . Is it time to lower the recommended screening age for colorectal cancer? J Am Coll Surg. 2011;213:352‐361. 21737316 10.1016/j.jamcollsurg.2011.04.033

[5] Bailey CE , Hu CY , You YN , et al. Increasing disparities in the age‐related incidences of colon and rectal cancers in the United States, 1975–2010. JAMA Surg. 2015;150:17‐22. 25372703 10.1001/jamasurg.2014.1756 PMC4666003

[6] Tawadros PS , Paquette IM , Hanly AM , Mellgren AF , Rothenberger DA , Madoff RD . Adenocarcinoma of the rectum in patients under age 40 is increasing: impact of signet‐ring cell histology. Dis Colon Rectum. 2015;58:474‐478. 25850833 10.1097/DCR.0000000000000318

[7] Mauri G , Sartore‐Bianchi A , Russo AG , Marsoni S , Bardelli A , Siena S . Early‐onset colorectal cancer in young individuals. Mol Oncol. 2019;13:109‐131. 30520562 10.1002/1878-0261.12417 PMC6360363

[8] Slattery ML , Boucher KM , Caan BJ , Potter JD , Ma KN . Eating patterns and risk of colon cancer. Am J Epidemiol. 1998;148:4‐16. 9663397 10.1093/aje/148.1.4-a

[9] Liang JT , Huang KC , Cheng AL , Jeng YM , Wu MS , Wang SM . Clinicopathological and molecular biological features of colorectal cancer in patients less than 40 years of age. Br J Surg. 2003;90:205‐214. 12555297 10.1002/bjs.4015

[10] Hill DA , Furman WL , Billups CA , et al. Colorectal carcinoma in childhood and adolescence: a clinicopathologic review. J Clin Oncol. 2007;25:5808‐5814. 18089879 10.1200/JCO.2007.12.6102

[11] Lieu CH , Golemis EA , Serebriiskii IG , et al. Comprehensive genomic landscapes in early and later onset colorectal cancer. Clin Cancer Res. 2019;25:5852‐5858. 31243121 10.1158/1078-0432.CCR-19-0899 PMC6774873

[12] Holowatyj AN , Gigic B , Herpel E , et al. Distinct Molecular phenotype of sporadic colorectal cancers among young patients based on multiomics analysis. Gastroenterology. 2020;158(1155–1158):e1152. 10.1053/j.gastro.2019.11.012 PMC7291587 31730769

[13] American Cancer Society . Colorectal cancer rates rise in younger adults; 2020. https://www.cancer.org/latest‐news/colorectal‐cancer‐rates‐rise‐in‐younger‐adults.html.

[14] van der Geest LG , Lam‐Boer J , Koopman M , Verhoef C , Elferink MA , de Wilt JH . Nationwide trends in incidence, treatment and survival of colorectal cancer patients with synchronous metastases. Clin Exp Metastasis. 2015;32:457‐465. 25899064 10.1007/s10585-015-9719-0

[15] Yang Q , Liao F , Huang Y , et al. Longterm effects of palliative local treatment of incurable metastatic lesions in colorectal cancer patients. Oncotarget. 2016;7:21034‐21045. 26992234 10.18632/oncotarget.8090 PMC4991510

[16] Raoof M , Haye S , Ituarte PHG , Fong Y . Liver resection improves survival in colorectal cancer patients: causal‐effects from population‐level instrumental variable analysis. Ann Surg. 2019;270:692‐700. 31478979 10.1097/SLA.0000000000003485

[17] Fenton HM , Taylor JC , Lodge JPA , et al. Variation in the use of resection for colorectal cancer liver metastases. Ann Surg. 2019;270:892‐898. 31567507 10.1097/SLA.0000000000003534 PMC6867670

[18] National Cancer Institute Surveillance, Epidemiology and End Results; Program Overview of the SEER Program . National cancer institute. surveillance, epidemiology, and end results program: SEER*Stat Database: SEER 18 Registries Custom Data (with additional treatment fields), Nov 2017 submission (1973‐2015 varying). 2020.

[19] Temple LK , Hsieh L , Wong WD , Saltz L , Schrag D . Use of surgery among elderly patients with stage IV colorectal cancer. J Clin Oncol. 2004;22:3475‐3484. 15337795 10.1200/JCO.2004.10.218

[20] Cook AD , Single R , McCahill LE . Surgical resection of primary tumors in patients who present with stage IV colorectal cancer: an analysis of surveillance, epidemiology, and end results data, 1988 to 2000. Ann Surg Oncol. 2005;12:637‐645. 15965730 10.1245/ASO.2005.06.012

[21] Venderbosch S , de Wilt JH , Teerenstra S , et al. Prognostic value of resection of primary tumor in patients with stage IV colorectal cancer: retrospective analysis of two randomized studies and a review of the literature. Ann Surg Oncol. 2011;18:3252‐3260. 21822557 10.1245/s10434-011-1951-5 PMC3192274

[22] van Rooijen KL , Shi Q , Goey KKH , et al. Prognostic value of primary tumour resection in synchronous metastatic colorectal cancer: Individual patient data analysis of first‐line randomised trials from the ARCAD database. Eur J Cancer. 2018;91:99–106. 29353165 10.1016/j.ejca.2017.12.014

[23] Stelzner S , Radulova‐Mauersberger O , Zschuppe E , et al. Prognosis in patients with synchronous colorectal cancer metastases after complete resection of the primary tumor and the metastases. J Surg Oncol. 2019;120:438‐445. 31168858 10.1002/jso.25578

[24] Siebenhuner AR , Guller U , Warschkow R . Population‐based SEER analysis of survival in colorectal cancer patients with or without resection of lung and liver metastases. BMC Cancer. 2020;20:246. 32293337 10.1186/s12885-020-6710-1 PMC7092492

[25] Poultsides GA , Servais EL , Saltz LB , et al. Outcome of primary tumor in patients with synchronous stage IV colorectal cancer receiving combination chemotherapy without surgery as initial treatment. J Clin Oncol. 2009;27:3379‐3384. 19487380 10.1200/JCO.2008.20.9817 PMC3646319

[26] Moritani K , Kanemitsu Y , Shida D , et al. A randomized controlled trial comparing primary tumour resection plus chemotherapy with chemotherapy alone in incurable stage IV colorectal cancer: JCOG1007 (iPACS study). Jpn J Clin Oncol. 2020;50:89‐93. 31829404 10.1093/jjco/hyz173 PMC6978626

[27] Gulack BC , Nussbaum DP , Keenan JE , et al. Surgical resection of the primary tumor in stage IV colorectal cancer without metastasectomy is associated with improved overall survival compared with chemotherapy/radiation therapy alone. Dis Colon Rectum. 2016;59:299‐305. 26953988 10.1097/DCR.0000000000000546 PMC4785825

[28] Comito T , Cozzi L , Clerici E , et al. Stereotactic Ablative Radiotherapy (SABR) in inoperable oligometastatic disease from colorectal cancer: a safe and effective approach. BMC Cancer. 2014;14:619. 25163798 10.1186/1471-2407-14-619 PMC4165935

[29] Jung J , Song SY , Kim JH , et al. Clinical efficacy of stereotactic ablative radiotherapy for lung metastases arising from colorectal cancer. Radiat Oncol. 2015;10:238. 26588896 10.1186/s13014-015-0546-x PMC4654895

[30] Ghiringhelli F , Hennequin A , Drouillard A , Lepage C , Faivre J , Bouvier AM . Epidemiology and prognosis of synchronous and metachronous colon cancer metastases: a French population‐based study. Dig Liver Dis. 2014;46:854‐858. 24908575 10.1016/j.dld.2014.05.011

[31] Venook AP , Niedzwiecki D , Lenz HJ , et al. effect of first‐line chemotherapy combined with cetuximab or bevacizumab on overall survival in patients with KRAS wild‐type advanced or metastatic colorectal cancer: a randomized clinical trial. JAMA. 2017;317:2392‐2401. 28632865 10.1001/jama.2017.7105 PMC5545896

[32] Cremolini C , Loupakis F , Antoniotti C , et al. FOLFOXIRI plus bevacizumab versus FOLFIRI plus bevacizumab as first‐line treatment of patients with metastatic colorectal cancer: updated overall survival and molecular subgroup analyses of the open‐label, phase 3 TRIBE study. Lancet Oncol. 2015;16:1306‐1315. 26338525 10.1016/S1470-2045(15)00122-9

[33] Xu J , Ma T , Ye Y , et al. Surgery on primary tumor shows survival benefit in selected stage IV colon cancer patients: A real‐world study based on SEER database. J Cancer. 2020;11:3567‐3579. 32284753 10.7150/jca.43518 PMC7150453

[34] Yi C , Li J , Tang F , et al. Is primary tumor excision and specific metastases sites resection associated with improved survival in stage colorectal cancer? Results from SEER database analysis. Am Surg. 2020;86:499‐507. 32684032 10.1177/0003134820919729

[35] Ciombor KK , Bekaii‐Saab T . A comprehensive review of sequencing and combination strategies of targeted agents in metastatic colorectal cancer. Oncologist. 2018;23:25‐34. 29021377 10.1634/theoncologist.2017-0203 PMC5759820

[36] Brudvik KW , Jones RP , Giuliante F , et al. RAS mutation clinical risk score to predict survival after resection of colorectal liver metastases. Ann Surg. 2019;269:120‐126. 28549012 10.1097/SLA.0000000000002319

[37] Gong J , Cho M , Fakih M . RAS and BRAF in metastatic colorectal cancer management. J Gastrointest Oncol. 2016;7:687‐704. 27747083 10.21037/jgo.2016.06.12 PMC5056249

[38] Venderbosch S , Nagtegaal ID , Maughan TS , et al. Mismatch repair status and BRAF mutation status in metastatic colorectal cancer patients: a pooled analysis of the CAIRO, CAIRO2, COIN, and FOCUS studies. Clin Cancer Res. 2014;20:5322‐5330. 25139339 10.1158/1078-0432.CCR-14-0332 PMC4201568

[39] Le DT , Uram JN , Wang H , et al. PD‐1 blockade in tumors with mismatch‐repair deficiency. N Engl J Med. 2015;372:2509‐2520. 26028255 10.1056/NEJMoa1500596 PMC4481136

